# Case Report: A Chinese child with Barth syndrome caused by a novel *TAFAZZIN* mutation

**DOI:** 10.3389/fcvm.2024.1465912

**Published:** 2024-09-06

**Authors:** Mingxuan Che, Fuhai Li, Yaning Jia, Qingzheng Liu, Jian Hu, Jidong Zhang, Shiguo Liu

**Affiliations:** ^1^ Cardiovascular Medicine Department, The Affiliated Hospital of Qingdao University, Qingdao, China; ^2^ Medical Genetic Department, The Affiliated Hospital of Qingdao University, Qingdao, China; ^3^ Prenatal Diagnosis Center, The Affiliated Hospital of Qingdao University, Qingdao, China

**Keywords:** Barth syndrome, cardiomyopathy, tafazzin, rare x-linked disease, neutropenia

## Abstract

Barth syndrome (BTHS) is a rare X-linked recessive genetic disorder characterized by a broad spectrum of clinical features including cardiomyopathy, skeletal myopathy, neutropenia, growth delay, and 3-methylglutaconic aciduria. This disease is caused by loss-of-function mutations in the *TAFAZZIN* gene located on chromosome Xq28, resulting in cardiolipin deficiency. Most patients are diagnosed in childhood, and the mortality rate is highest in the early years. We report a case of acute, life-threatening metabolic decompensation occurring one day after birth. A novel *TAFAZZIN* splice site mutation was identified in the patient, marking the first reported case of such a mutation in BTHS identified in China. The report aims to expand our understanding of the spectrum of *TAFAZZIN* mutations in BTHS.

## 1 Introduction

BTHS (MIM 302060) was initially identified as a rare X-linked recessive genetic disorder primarily affecting the mitochondrial function in neutrophils, as well as cardiac and skeletal muscles. The syndrome is characterized by cardiomyopathy, neutropenia, developmental delays, skeletal muscle dysfunction, and 3-methylglutaconic aciduria (1). The causative factor of BTHS has been traced to loss-of-function mutations in the *TAFAZZIN* gene located on the Xq28 region of the X chromosome (2). This study reports a Chinese pediatric case of BTHS, which is the first reported case of this specific novel splice site mutation. This discovery enhances our understanding of the molecular mechanisms underlying the disease.

## 2 Case presentation

**Figure 1:**
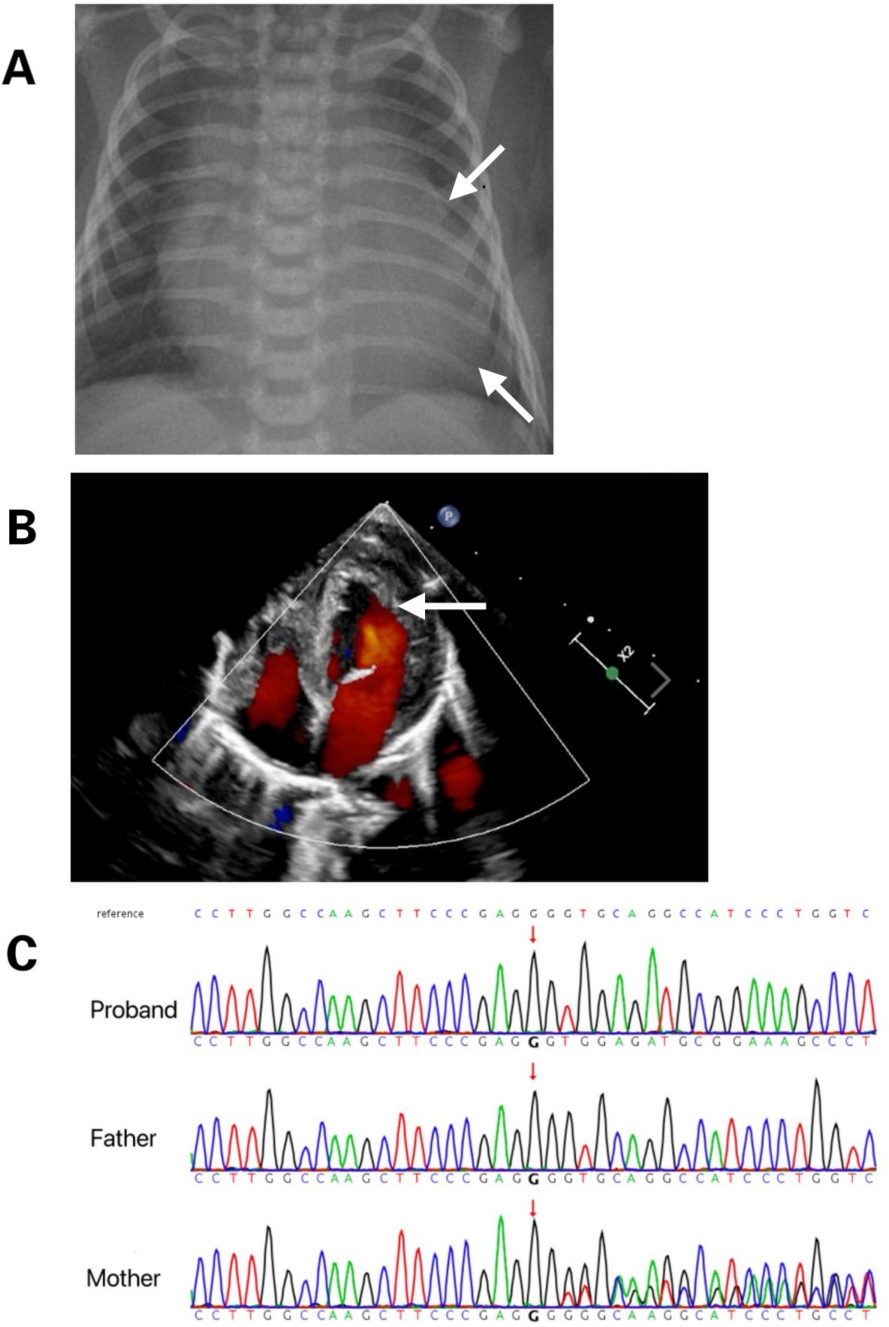
**(A)** Chest radiograph displays marked cardiomegaly. **(B)** Cardiac ultrasound reveals myocardial hypertrophy. **(C)** Tafazzin sequencing electropherogram displays that the patient is hemizygous for the in-frame deletion, c.778- 30_778del (arrow). The variant is not detected in his mother, indicating that it was a *de novo* mutation.

The male neonate was delivered via cesarean section at 36 weeks of gestation due to fetal cardiac anomalies and umbilical cord entanglement. He weighed 2,780 g at birth, with Apgar scores of 9 at 1, 5, and 10 min. His condition deteriorated rapidly after delivery, with severe cyanosis, frothing, and reduced vitality. Initial vital signs demonstrated no fever, low blood pressure (65/29 mmHg), tachycardia (heart rate 128–152 beats/min), or rapid breathing (respiratory rate 52–62 breaths/min), with oxygen saturation at 91%–95%. Chest radiography revealed cardiomegaly (Figure 1A). Concurrent echocardiography showed left atrial and ventricular enlargement with thickening of the ventricular wall and septum, with a left ventricular internal diameter of 1.9 cm and a wall thickness of 0.5 cm. The left ventricular ejection fraction was 36%, indicating decreased contractility, but there was no noncompaction of the LV myocardium (Figure 1B). Blood tests revealed metabolic acidosis (pH 7.21, PCO2 59 mmHg, HCO3–0.60 mmol/L), coagulation abnormalities (activated partial thromboplastin time 3.12), creatine kinase levels at 438 U/L, and brain natriuretic peptide levels at 15,638 pg/ml, with normal troponin levels. The neutrophil count was 1.36*10^9^/L. Based on these clinical manifestations, BTHS was suspected. Treatment included continuous low-flow nasal cannula oxygen and anti-infective therapy with piperacillin sodium, tazobactam sodium, sodium creatine phosphate, and oral digoxin to enhance myocardial function. The patient's cardiac function improved significantly, and he was eventually discharged. Subsequent whole-exome sequencing was performed to identify mutations in the couple and their children, revealing a novel DNA splicing site mutation in intron 10 of *TAFAZZIN* (c.778 -30_778del) (Figure 1C). The mutation was inherited from a mother with no cardiac disease history. According to the American College of Medical Genetics and Genomics criteria (Pathogenic very strong 1 and Moderate 2), this novel variant was likely pathogenic.

## 3 Discussion

BTHS is an X-linked recessive genetic disorder first described by Barth et al. (3) characterized by cardiomyopathy, skeletal myopathy, neutropenia, growth delay, and increased urinary excretion of 3-methylglutaconic acid (3). Additional features include dilation, hypertrophy, and incomplete compaction of the left ventricle, leading to congestive heart failure. Early studies indicated high mortality rates in infants and children (4). Molecular genetic testing for mutations in *TAFAZZIN* has been used to diagnose BTHS, with over 120 pathogenic mutations identified across all 11 exons and introns of the gene (5). Nicola et al. described younger cousins with dilated cardiomyopathy including myocardial thickening, excessive trabeculation, mild-to-moderate mitral valve insufficiency, and impaired left ventricular contractile function with an ejection fraction of 38%. Older cousins exhibited mild clinical features. A hemizygous DNA splice site mutation (c.777+1G>A) was identified in intron 10 of *TAFAZZIN* (6) among these cousins. TAE et al. also reported a 13-month-old boy with refractory heart failure attributed to dilated cardiomyopathy due to hemi-frame shift deletion of nine amino acids within exon 10 of *TAFAZZIN* (c.725_751del, p.Pro242_Glu250del) (7). Similar to the results of our study, these reports indicate that *TAFAZZIN* mutations may affect cardiac metabolism and dysfunction. Laure et al. reported the first confirmed case of BTHS in a female patient who experienced severe heart failure at one month old diagnosed with dilated, hypo contractile, and hypertrophic cardiomyopathy, with incomplete contraction of the left ventricle (8). Then, a large deletion encompassing the first five exons of *TAFAZZIN* was confirmed through genetic analysis. BTHS is caused by mutations in *TAFAZZIN* located on chromosome Xq28, which encodes tafazzin, a protein involved in the remodeling of cardiolipin, a phospholipid vital for mitochondrial membrane integrity. Tafazzin, a phospholipid transacylase, is involved in the remodeling of phosphatidylglycerol and cardiolipin within the inner mitochondrial membrane. Defects in tafazzin enzyme activity lead to cardiolipin loss, disrupting the stability of respiratory chain supercomplexes, and impairing electron transport chain function (9–11). Recent studies have demonstrated that the genetic background of a mouse model of BTHS influenced the phenotypic expression of tafazzin, potentially altering mitochondrial quality control (12). A new mutation identified in China, a DNA splicing site mutation in intron 10 of *TAFAZZIN* (c.778-30_778del), aids in establishing the genotype-phenotype relationship of tafazzin induced BTHS and improves our understanding of its function.

## 4 Conclusions

This report shows a splicing site mutation in intron 10 of *TAFAZZIN* (c.778-30_778del), which is the first genetically confirmed case of BTHS in a Chinese child.

## Data Availability

The original contributions presented in the study are included in the article/Supplementary Material, further inquiries can be directed to the corresponding author.
